# A review of barriers and facilitators to mammography in Asian women

**DOI:** 10.3332/ecancer.2020.1146

**Published:** 2020-11-23

**Authors:** Zohre Momenimovahed, Azita Tiznobaik, Safoura Taheri, Soheil Hassanipour, Hamid Salehiniya

**Affiliations:** 1 Qom University of Medical Sciences, Qom, Iran; 2 Hamedan University of Medical Sciences, Hamedan, Iran; 3 Ilam University of Medical Sciences, Ilam, Iran; 4 Gastrointestinal and Liver Diseases Research Center, Guilan University of Medical Sciences, Rasht, Iran; 5 Social Determinants of Health Research Center, Birjand University of Medical Sciences, Birjand, Iran

**Keywords:** breast cancer, screening, mammography, barriers, facilitators, Asia

## Abstract

**Aim:**

Breast cancer is the most common cancer among women in Asia and one of the major health problems in most countries in the region. Despite extensive advances in treatment, early diagnosis is one of the main methods for increasing the survival rate. It is necessary to evaluate the barriers and facilitators of screening in different societies. This study was conducted to identify the barriers and facilitators of mammography in Asia.

**Materials and methods:**

To identify the barriers and facilitators of mammography in Asia, a comprehensive search was carried out in databases such as Medline, Web of Science Core Collection and Scopus using keywords, including breast cancer, screening, mammography, barriers, facilitators and the names of each Asian country, as well as a combination of these keywords were used to help the search. Full-text, English language and original articles were included in this study.

**Results:**

In total, 71 articles were entered into the study. The findings of this study revealed various barriers to mammography in Asian women, including knowledge, sociodemographic factors, cost and insurance, cultural factors, belief, attitude and feeling, fear, pain and embarrassment, self-efficacy, religious, psychological factors, time constraint, fatalism, professional recommendations, communication, social support and access. Also, knowledge, attitude and belief, perceived risk, professional and social factors were found to be facilitators of mammography.

**Conclusion:**

Knowing the barriers and facilitators to access mammography is the first step in the development of a successful screening program. Awareness and various personal, professional and social factors have emerged as the major barriers to access to mammography in most Asian countries.

## Introduction

Asia is the largest and most populous continent in the world. It also has the most socio-economic diversity between the countries [1]. Breast cancer is the most common cancer among women in Asia and one of the major health problems in most countries in the region [2]. In 2012, about 1.67 million new cases of breast cancer were identified worldwide, accounting for 25% of all cancer cases [3]. Nearly 24% of all breast cancers occur in Asia-Pacific, and China, Japan and Indonesia have the highest prevalence of breast cancer in Asia [2]. The Age-Standardised Rate (ASR) of breast cancer varies widely across Asia from 4.6 in Bhutan to 65.7 in Singapore [4]. Despite recent advances in the diagnosis and treatment of breast cancer, it remains one of the major health challenges in many parts of the world, including Asia [5].

Early detection and effective treatment are two main factors that reduce morbidity and mortality of breast cancer. However, despite extensive advances in treatment, early diagnosis is one of the main methods for increasing the survival rate [6]. Self-examination, clinical examination and mammography of breasts are effective methods of breast cancer screening, and in recent years, mammography has become the standard method of breast cancer screening [7]. The American Cancer Society recommends that breast cancer screening for women at average risk begin at age 45 and continues until they are in good health and have a life expectancy of 10 years or more [8]. In younger women, mammography is recommended only for people with a family history, a history of chest radiation and a history of benign breast disease, but if they have this cancer, their survival rate is lower and therefore knowledge of breast cancer and screening methods is very important in this age group [9]. However, despite the high effectiveness of mammography in reducing mortality and morbidity associated with breast cancer, the screening rate is very low in many areas [10].

Awareness of the factors associated with mammography is the first step in improving screening rates in different populations. Knowledge, attitudes and beliefs related to breast cancer screening are among the most important factors that influence the cultural, social and economic contexts of each country and region. So, it is necessary to evaluate the barriers and facilitators of screening in different societies before designing and implementing a strategy to achieve universal coverage of breast cancer screening programmes. Studies show that ethnicity influences the use of mammography by influencing culture and lifestyle. Therefore, the use of screening and preventive methods in many people in one region will not change even if they migrate to another country [11, 12]. Thus, this study was conducted to identify the barriers and facilitators of mammography in Asia.

## Materials and methods

### Search strategy

An extensive search was conducted in databases such as Medline, Web of Science Core Collection (Indexes = SCI-EXPANDED, SSCI, A & HCI Timespan) and Scopus to find articles published from beginning to November 2019. Keywords such as breast cancer, screening, mammography, barriers, facilitators and the names of each Asian country, as well as a combination of these keywords, were used to help the search. Then, to ensure the comprehensiveness of the search, manual searches in valid journals were also performed, followed by manual searches in the references of full-text articles as well as references of systematic reviews. All recovered articles were entered into Endnote X7 within a database.

### Inclusion criteria

Inclusion criteria in this study were: being an original study, being conducted on breast cancer screening with mammography, referring to breast cancer screening factors, being full-text qualitative or quantitative articles in English and using the keywords in their title or abstracts. No limitations were applied in this study in order to not to limit the results.

### Exclusion criteria

Being conducted on mammography after breast cancer diagnosis, not having the full text and using other screening methods than mammography, as well as commentaries, editorials, conference abstracts, opinion statements, practice guidelines and case series or case reports were the exclusion criteria.

### Data extraction

The article search was carried out by one of the researchers. Two other researchers independently evaluated the inclusion and exclusion criteria of the articles based on their title and abstract. After excluding articles that did not meet the inclusion criteria, the full texts of the remaining articles were reviewed and included in this study. In the next step, the study results were extracted qualitatively.

## Results

### Specifications of the accepted studies

In the initial search, 378 articles were obtained from databases and 16 articles were extracted by manual search. After removing duplicate articles by Endnote software, 342 articles were selected for review. After reviewing the title and abstract, 251 articles that were not relevant to the purpose of the study and did not meet the study criteria were excluded. Also, 20 articles were excluded for scientific reasons (duplicate: 3, not available full text: 5, barriers not defined: 3, inadequate results: 6, not in English: 3). Finally, 71 articles published during 1994–2019 were selected for the review (Flowchart 1).

### Specifications of the accepted studies

Overall, 61 articles had been carried out by the quantitative method and 10 articles had been carried out by the qualitative method. Screening methods were different between the studies. For example; 18 studies had been using breast self-examination, clinical examination and mammography, and 41 articles had only been using the mammography method. The number of samples in the studies varied from 20 (13) to 53513 (14), (Table 1).

### Barriers of mammography

Barriers and facilitators of mammography in Asian women are shown in Table 2.

### 1. Personal factors

#### 1-1. Knowledge

Knowledge and awareness of people is one of the key elements of success in any screening program. Awareness about the need for mammography at the asymptomatic stage is a key factor in program coverage and result follow-up. Lack of awareness is one of the most prominent barriers to screening in different countries [15, 16, 23, 24, 34, 58, 59, 61, 62, 83]. Low level of awareness about breast cancer and its diagnostic methods [16, 25, 35, 61], advantages of early detection of breast cancer, the necessity and importance of screening, the time of screening [25], knowledge of how to perform screening, clinics available in the city and consequences of late detection of breast cancer justify the low prevalence of mammography screening in many Asian countries [26, 36–38, 61, 63, 64, 84]. In a study in Turkey, those who had information about mammography were eight times more likely to perform screening than those who did not have information [27], (OR ¼ 8.83, 95% CI: 3.27, 23.84). In a study in Bangladesh, 40.1% of people referred to the lack of awareness about the necessity of screening as a reason for low screening rate [63]. The relationship between knowledge, awareness and performing mammography is influenced by cultural attitudes and barriers [84]. Studies show that a low level of knowledge in many countries stems from their traditional culture. People’s reluctance to talk about sensitive issues such as breast cancer has led to a decrease in screening behaviours among them [28, 85, 86].

#### 1-2. Sociodemographic factors

Differences in the use of screening services with regard to demographic characteristics of individuals have been well examined in different studies. Some of these characteristics such as age [29, 30, 39, 84, 87], level of education [41, 42, 63, 87], socioeconomic status [58, 63, 65, 87] and employment status [43, 84] influence people’s attitude and level of screening utilisation. Also, menopausal age, hysterectomy, menarche age [42], history of oral contraceptive pill (OCP) use, marital status [41, 43, 87], body mass index (BMI) [43], urban life and place of residence [39, 41], alcohol consumption and smoking [87] have been linked to breast cancer screening. Some Chinese women have stated that mammography is an age-dependent method and older people do not need it [36]. In a study in China, married women had the highest breast cancer screening rate (24.3%; 95% CI, 21.4%–27.2%) and women who had never been married had the lowest screening rate (7.3%; 95% CI, 5.6%–8.9%, *p* < 0.001) [14]. According to this study, women over 60 years of age had the lowest screening rate for breast cancer. In this study, women younger than 30 years of age were 2.2 times more likely to perform mammography (95% CI, 1.8–2.7, *p* < 0.001) than women younger than 30 years. This rate was also 4.8 times higher in the age group of 30–39 years (95% CI, 4.0–5.8, *p* < 0.001) and 4.8 times higher in the age group of 40–49 years (95% CI, 4.0–5.7, *p* < 0.001) [14]. The study of Gang et al. showed that people without children or with only one child were more likely to seek screening (OR = 4.879; 95% CI (1.835–12.976)) [42]. However, many sociodemographic factors have been found to be major obstacles to mammography in many studies, some researches show that people’s financial status [41, 44], employment status [27, 39], age [42, 45, 46], income [27], family history of breast cancer [27, 46], education level [82], marital status [27, 45], menopause [45], use of OCP [42] and high BMI [42]( are not correlated to mammography.

#### 1-3. Cost and insurance

Many hypotheses have been put forward regarding the causes of noncompliance with screening programs, but one of the most tangible barriers is the cost of screening [15, 21, 22, 26, 34, 57, 81], which is more prominent in young, unemployed and low-educated people [20, 23, 25, 88, 89]. Although in many countries this barrier has been moderated by insurance coverage, the high cost of screening and lack of insurance coverage are among the factors that prevent people from performing mammography [25, 27, 39, 43]. Insurance in any form, whether public or private, affects people’s decision to weather pay for mammography or not [19, 33]. Some Chinese women point out that their insurance contract will be terminated after their retirement, which makes it difficult for them to afford the high costs of mammography [36]. In a study in Turkey, uninsured people were almost half the time less likely to participate in screening programs than others (2.76, 95% CI 1.24, 6.17) [27]. However, the relationship between insurance coverage and mammography is not significant in some studies. Researchers’ experience in Saudi Arabia has shown that cost does not play an important role in the low screening rate in this country because screening is free of charge [80]. A study in Iran did not identify insurance as one of the factors influencing mammography and revealed that access to insurance coverage did not play a role in one’s decision to repeat mammography [79]. This could be due to the low share of insurance in supporting people to carry out screening programs.

#### 1-4. Cultural factors

Culture plays a major role in shaping people’s attitudes towards health and disease [19], as well as women’s participation in screening programs [13, 16, 25, 78]. The experience of health policymakers in Saudi Arabia has shown that, despite free mammography screening, women’s participation in the program is still poor, and this is rooted in some traditions and customs [24, 80]. The inconsistent results between different studies have led the researchers to pay more attention to cultural barriers. Given the structure of the family in many countries and gender roles, men make important decisions in all matters and women alone cannot make a decision on screening [25, 56, 59]. Husband’s disagreement and restriction of women’s driving or travel of unmarried women due to specific customs in some Arab countries [16, 18] are other cultural barriers that overshadow the mammography rate.

#### 1-5. Beliefs, attitude and feeling

Since there is a strong relationship between beliefs and practice, one’s attitudes and beliefs can play a role in screening for breast cancer [19, 84]. Many people around the world believe that there is no reason to do mammography in the absence of symptoms [19, 23–26, 34, 36, 55, 63, 64, 76, 77], as mammography can also be risky due to its radiation [32, 58, 90]. Thus, they believe that mammography should be limited to the time when the symptoms appear [43]. This view is caused by poor awareness and a lack of information on mammography at the asymptomatic stage [43]. Some believe that breast cancer occurs in people with a family history of cancer and risk factors [36, 75] or in people with an unhealthy lifestyle, following the consumption of fast foods, chemicals, stress and anxiety, and therefore people with a healthy lifestyle do not need to perform mammography [61]. Some Asian women believe that breast examination is sufficient to detect mammary masses and mammography is only needed after the diagnosis. They also believe that cancer is asymptomatic and can only be diagnosed in advanced stages [61]. In addition to misconceptions, doubts about the effectiveness of mammography can also act as a barrier to mammography, as many Asian women believe that mammography is not as effective in some Asian societies as it is in Western societies [31]. Some other Asian women believe that cancer is a disease of old age, so young people do not need to be screened [24, 77] and also early diagnosis cannot play a significant role in recovery from cancer [54]. In addition, some women believe that after they have been diagnosed with breast cancer, their mental focus has only been on their illness, and this puts pressure on their family, children, jobs and relationships with their spouses [91]. In Pakistan, cancer is considered a contagious disease, which is transmitted from person to person in contact with those affected. This causes some Pakistani women to be reluctant to diagnose cancer early and therefore, many of them are diagnosed with advanced stages of cancer [91]. Others’ unpleasant experience with mammography reduces the willingness of those around them to perform this screening test, thus it acts as a barrier to mammography [64, 74].

#### 1-6. Fear, pain and embarrassment

Fear is the most common psychological barrier that has been considered by most studies [30, 60, 73]. Fear of people in various ways can prevent them from performing mammography. Fear of breast cancer diagnosis [23–26, 30, 32, 34, 53, 54, 62, 72–74, 76, 77, 83], fear of pain [26, 39, 52, 62, 75, 83] and fear of shame [23] were three common types of fear in various studies. Fear of breast cancer diagnosis due to the fear of treatment [61], fear of radiation [23, 25, 26, 31, 36, 61, 62, 72, 77], fear of device-induced cancer [20, 31, 53], fear of the consequences of breast cancer such as chemotherapy, pain (61), cancer-related death [25, 61], medical costs, mastectomy and its following sense of nonattractiveness [61] and eventually fear of ruining family life [73] are other types of fear that women experience during mammography. Painful mammography can affect screening behaviour in the absence of symptoms of breast cancer [20] and prevent women from performing mammography [17, 23, 25, 37].

One of the fears mentioned in some studies is the fear of stigma. Since cancer is a stigmatised disease, many people are afraid of getting it and believe that the diagnosis of cancer is not socially accepted. They believe that although cancer is a fatal disease, the diagnosis of breast cancer can result in the disintegration of life and divorce [16, 54, 61, 71, 76]. However, some studies have suggested that fear of disease progression and mastectomy can be a facilitating factor in breast cancer screening [78].

#### 1-7. Self-efficacy

Self-efficacy in mammography means confidence and the ability to overcome the barriers of screening. According to studies, self-efficacy [26, 60, 70], chronic diseases, disability and visual, hearing, and motor problems are some of the barriers to mammography [40]. Chronic disease weakens people’s view of mammography, so they do not prioritise mammography [74].

#### 1-8. Religions

There is a long history of research on the relationship between religion and preventive activities, and religious reasons have been found to be one of the barriers to cancer screening in Muslim countries [25, 51, 63, 81]. The relationship between religion and breast cancer screening can be examined from different aspects. First, the discrimination that religious people face in receiving care and services can act as a barrier to screening. On the other hand, people with religious mechanisms that help them cope with illnesses and problems are less likely to be screened [92]. The lack of female physicians in some countries is a major obstacle to women’s decision to have mammography [24]. In addition, some women believe that although God has commanded his servants to take care of their health, trust in God and the Holy Quran acts as a preventive measure against diseases [83]. These people consider spirituality and God-ordained prayer as the ways of fighting cancer and do not consider mammography necessary [74]. On the one hand, some believe that breast cancer can be a punishment or a gift from God to human beings [54, 74].

#### 1-9. Psychological factors

Asian women have a different sense of body-image. Many of these people consider their body to be completely private and refuse to disclose their private parts to strangers even to service providers [17, 20, 62]. These people believe that breast-related issues are a taboo subject and it is a shame to talk about breasts, so they question the need for screening [17, 20, 21, 25, 31, 37, 52, 53, 58, 63, 72]. This barrier is much more pronounced when a male staff performs the mammography [25, 32, 59, 76]. The most common reason for not having mammography in a study in India was the shame and discomfort that come from revealing the breasts to someone else [32]. Also, single women were more embarrassed than other women to have a gynaecologist visit and perform mammography [74].

#### 1-10. Time constraint

People’s priorities in life differ from each other. Many women in Asia believe that there are more important issues in their lives than mammography, and pursuing their own health issues keeps them away from caring for their families. In addition, family activities take up most of their time and leave them with no time for screening and follow-up [20–23, 25, 26, 39, 43, 52, 57, 61, 77]. Chinese women believe that mammography is a time-consuming process, and mental distractions discourage one from performing mammography (36). Also, out of home employment is often high among women in their 40s, when the risk of breast cancer is rapidly increasing. Meanwhile, balancing work and health care is difficult [43]. Some studies show that social, occupational and intellectual problems [26], multiple social, family and professional roles [17], lack of time due to other responsibilities, such as caring for children, and employment are among other barriers to breast cancer screening [24, 25, 60, 83].

#### 1-11. Fatalism

Cancer fatalism is a set of attitudes and behaviours that suggest cancer screening and treatment is futile and disease prevention is beyond human control [17, 76, 93–95]. Researchers believe that the link between cancer and fatalism results from poverty, low education and old age [95, 96], and is also higher in people with no history of mammography [69]. Women in many Asian countries believe that death and life are our fate, which cannot be changed. If cancer and the resulting death are our destiny and there is no way out, screening tests are no longer required [15, 17, 25, 26, 54, 60, 74, 83]. Increased fatalism reduces fear of breast cancer and decreases motivation for mammography [68].

### 2. Professional factors

#### 2-1. Professional recommendations

Lack of physician’s recommendation is one of the main causes of noncompliance with mammography [19, 25–27, 37, 59, 64], because people believe that there is no need for a test unless the doctor advises to do so [25, 53]. In a study in Qatar, despite low awareness and poor participation in breast cancer screening, only a quarter of participants stated that their physician recommended screening [54]. Not trusting physicians’ diagnosis due to inadequate skills and diagnostic errors is another barrier to mammography, which is related to physicians [16, 61, 83]. Distrust of the physician results from the past positive and negative experiences [83].

#### 2-2. Communication

The role of effective communication in preventive measures is unknown. Ineffective communication of service providers has been identified as one of the barriers to mammography [26, 72]. Some people believe that service providers treat mammography indifferently and address women’s needs and concerns disrespectfully [25, 26]. In addition, not preserving privacy by these individuals discourages many women from performing mammography [58.

### 3. Social factors

#### 3-1. Social support

Social support, including family, friends, relatives and co-workers, can play an important role in promoting a positive view of preventive measures. Lack of support from family and friends [81], crowded clinics [25, 26], especially in public health centres, and shortage of health centres in proportion with population are among other barriers to mammography [61].

#### 3-2. Access

Limited access to regular health care sources and screening clinics has been identified as another reason for the low rate of breast cancer screening in some regions of Asia [25, 26, 60, 84]. However, in some studies, difficult access to mammography centres due to long distance and commuting problems was not identified as one of the major barriers to mammography [21, 36, 59, 67]. The results of another study questioned the role of access in screening. In Saudi Arabia, according to Bcheraoui’s study, screening centres are less than 8 kilometres away from people’s home, therefore local access cannot be an effective factor in performing mammography, and screening rates range from 0.0% and 0.6% in Al-Jawf and Al-Hudad up to 9.7% and 17.00% in Al Sharqia and Nijran [80], respectively.

**Facilitators of mammography**

### 1. Personal factors

#### 1-1. Knowledge, attitude and beliefs

Positive attitudes and beliefs about screening can play a role in encouraging people to perform mammography [26]. Many women refer to their responsibility for health [10, 16] and believe that when screening procedure affects health, you cannot ignore it [26]. Knowledge and awareness of women affect their attitudes towards mammography. Studies show that when women are aware of breast cancer and the importance of its timely diagnosis, their participation in mammography screening programs increases [27, 36, 75]. Level of education also plays an important role in performing breast cancer screening [16, 46, 50, 65]. In a study in China, the rate of breast cancer screening ranged from 9% (95% CI, 7.3%–10.8%) in people with less than 6 years of education to 36.6% (95% CI, 33.0%–40.2%) in people with over 12 years of education [14]. Self-efficacy is another individual factor associated with breast cancer screening [36, 49, 50]. According to researchers, higher self-efficacy and quality of life are associated with increased participation in the breast cancer screening program [65].

#### 1-2. Perceived risk

People’s perceived risk is a motivation for mammography. Studies show that perceived vulnerability facilitates mammography [39, 49, 84]. History of mammary problems such as pain, abnormal breast discharge and mammary abscess, masses and cysts can be an effective facilitator for mammography [25, 34, 39, 46, 48, 57, 66, 79]. In addition to individual history, family history of breast cancer also increases one’s motivation to perform mammography [26, 47]. A study in Iran showed that people with a family history of breast cancer were twice as likely to participate in breast cancer screening [39].

The preventive approach and the belief that a timely diagnosis of cancer can increase the chances of survival have been identified as one of the facilitators of mammography [15, 26, 34, 54]. Repeated use of complementary therapies in Japan encouraged people to perform mammography [47]. In addition, the perceived benefits of mammography in early detection and increased survival increase the rate of mammography [15]. Participants who perform regular mammography have the same fears as other people, but in many cases, they cope with these fears because of their perceived risk and severity of symptoms. They also think that early detection increases their chances of survival [15, 16, 81]. Self- and clinical examinations of the breast also lead people towards mammography [10, 36].

### 2. Professional factors

Regular medical visits [26, 27, 34] and physician’s recommendation [25, 27, 60, 75, 76, 84] are the strongest predictors of mammography in different countries.

### 3. Social factors

Recommendation of friends, family and relatives [26, 75, 81, 83], raising awareness of people by media and social networks [15, 25, 26] and the existence of supportive social context [16] play an important role in mammography screening. The positive experience of family and friends in mammography is one of the facilitators of breast cancer screening [50]. Social support is also one of the facilitators of breast cancer screening. Social support by inducing self-efficacy in people and overcoming the financial and psychological barriers will create a strong motivation for mammography [79]. Health-seeking behaviours have a social nature, which means that a person is more likely to perform mammography when he or she feels that it is important to their family and is supported by them [17]. Easier access to health services such as insurance [19, 27], higher number of screening centres and government subsidies to reduce the costs also play an important role in performing mammography [25, 26].

## Limitation

This study has some limitations that need to be addressed. Many studies have used self-reports about mammography, which can distort the results. On the other hand, the small sample size of some studies and also the use of convenient sampling, which reduces the generalisability of the results, are other limitations of this study. Although using the integrative review method due to the extensive search of articles, the power of the study increases, but because of the differences in the type of studies and multiple definitions, the analysis and comparison are difficult. Lack of classification of barriers by age as well as income and country is another limitation of this study. Finally, due to the identification of broader barriers, low-quality studies were not excluded from this study.

## Discussion

Mammography as a method of screening and diagnosing breast cancer has been introduced and available in different countries for many years. Although the results of some studies show that the reduction in breast cancer mortality is not only attributable to mammography [97–99] and therefore the recommendation for routine mammography requires further investigation [98], it is still an accepted screening method in the world.

Identifying barriers and facilitators of mammography is crucial in encouraging women to participate in this screening program. Early detection of breast cancer requires the awareness of women and healthcare professionals about the early signs of breast cancer and the benefits of early diagnosis [100]. Although awareness is one of the cornerstones of comprehensive coverage of screening programs [101], in many low- and middle-income countries, inadequate infrastructure and limited resources have prevented screening goals from being achieved [25, 26, 60, 84, 102]. In these countries, limited access and factors such as insufficient awareness, socioeconomic barriers and cultural challenges are associated with the presentation in the higher stages of the disease, a weaker prognosis and consequently high mortality [103, 104]. It seems that the first priority in reducing mortality in these countries is to develop the conditions and facilities for early diagnosis, followed by raising awareness and overcoming other barriers to mammography screening [103, 105].

## Conclusion

In the present study, the barriers and facilitators of breast cancer screening were studied. The findings of this study revealed various barriers to mammography in Asian women, including knowledge, sociodemographic factors, cost and insurance, cultural factors, belief, attitude and feeling, fear, pain and embarrassment, self-efficacy, religious, psychological factors, time constraint, fatalism, professional recommendations, communication, social support and access. Also, knowledge, attitude and belief, perceived risk, professional and social factors were found to be facilitators of mammography.

## Conflicts of interest

There are no conflicts of interest.

## Funding

None.

## Figures and Tables

**Flowchart 1.**
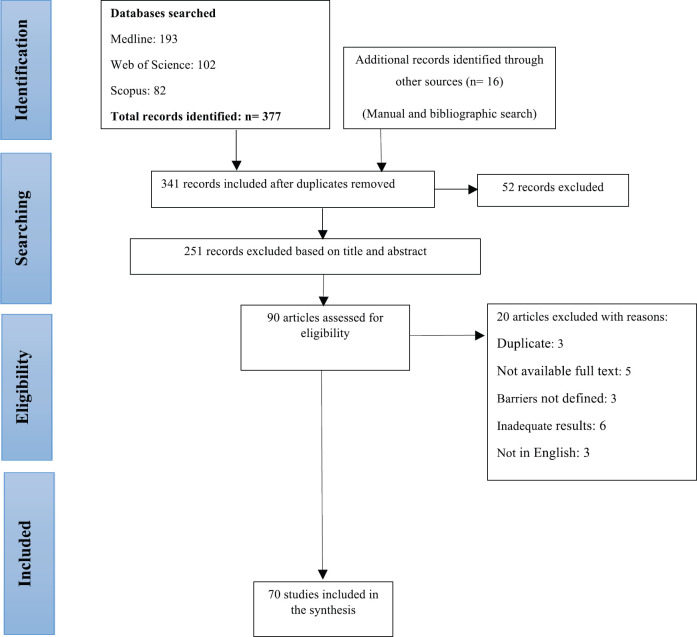
Included eligible studies in the review.

**Table 1.** Characteristic of included studies in the review.

Study characteristic	No (%) of the studies (*n* = 70)
Year 1994–1999 [15] 2000–2004 [16–22] 2005–2009 [23–33] 2010–2014 [13, 14, 34–57] 2015–2019 [58–82]	1 (1.4) 7 (10.0) 11 (15.71) 26 (36.61) 25 (35.71)
Participant number <50 [13, 15, 16, 26, 31, 60, 61, 74, 78] 50-<200 [19, 20, 22, 30, 32, 38, 49, 56, 70] 200-<500 [21, 23, 25, 34, 39, 42, 44, 46, 48, 50, 51, 53, 57–59, 64, 66, 68, 69, 71, 72, 79, 82] 500-<1,000 [17, 27, 28, 33, 36, 37, 52, 55, 73, 76, 77, 81] >1,000 [14, 18, 24, 29, 35, 40, 41, 43, 45, 47, 54, 62, 63, 65, 67, 75, 80]	9 (12.85) 9 (12.85) 23 (32.85) 12 (17.14) 17 (24.28)
Type of study Qualitative [13, 15, 16, 26, 31, 56, 60, 61, 74, 78] Quantitative [14, 17–25, 27–30, 32–55, 57–59, 62–73, 75–77, 79–82]	10 (14.29) 60 (85.71)
Country Afghanistan [56] Bangladesh [63] Emirates [16, 18] China [14, 15, 19, 21, 31, 32, 36, 42, 45, 47, 65] India [64] Iran [26, 39, 48–53, 55, 61, 66, 68–70, 74, 76, 79] Iraq [13, 71] Japan [43, 67] Jordan [20, 37, 81] Korea [22, 23, 30, 40, 41] Kuwait [60] Lebanon [72] Malaysia [17, 34, 38, 46, 75] Pakistan [59] Qatar [35, 54] Saudi [24, 28, 58, 62, 73, 77, 80] Singapore [57] Turkey [25, 27, 29, 44, 78, 82] Multi-region [33]	1 (1.42) 1 (1.42) 2 (2.85) 11 (15.71) 1 (1.42) 17 (24.28) 2 (2.85) 2 (2.85) 3 (4.28) 5 (7.14) 1 (1.42) 1 (1.42) 5 (7.14) 1 (1.42) 2 (2.85) 7 (10.0) 1 (1.42) 6 (8.57) 1 (1.42)
Setting Urban [13, 15–20, 23–28, 32, 34, 36, 38, 42–44, 46–48, 50–53, 55–61, 64, 66, 68–72, 74–76, 79] Rural (29, 65) Urban and rural [14, 30, 35, 37, 39–41, 45, 54, 62, 63, 67, 73, 77, 78, 80, 81] NM [21, 22, 31, 33, 49, 82]	45 (64.28) 2 (2.85) 17 (24.28) 6 (8.57)
Screening method Mammography [15–17, 20, 23, 30, 31, 33, 34, 36–39, 41, 42, 44, 47–53, 55, 57, 59–62, 64, 66, 67, 69, 70, 74–76, 79, 81, 82] Breast self-exam and mammography [26–29, 58, 77] Clinical and mammography [13, 14, 46, 56, 63, 73] Breast self-exam and mammography and CBE [18, 19, 21, 22, 24, 25, 32, 35, 40, 43, 45, 54, 65, 68, 71, 72, 78, 80]	40 (57.14) 6 (8.57) 6 (8.57) 18 (25.71)

**Table 2.** Barriers and facilitators of mammography in the review.

Category	Barriers	Facilitators
Personal factors	Knowledge Sociodemographic factors Cost and insurance Cultural factors Belief, attitude and feeling Fear, pain and embarrassment Self-efficacy Religious Psychological factors Time constraint Fatalism	Knowledge, attitude and beliefs Perceived risk
Professional factors	Professional recommendations Communication	
Social factors	Social support Access	
